# New interaction partners for Nek4.1 and Nek4.2 isoforms: from the DNA damage response to RNA splicing

**DOI:** 10.1186/s12953-015-0065-6

**Published:** 2015-02-26

**Authors:** Fernanda Luisa Basei, Gabriela Vaz Meirelles, Germanna Lima Righetto, Deivid Lucas dos Santos Migueleti, Juliana Helena Costa Smetana, Jörg Kobarg

**Affiliations:** Laboratório Nacional de Biociências, Centro Nacional de Pesquisa em Energia e Materiais, Rua Giuseppe Máximo Scolfaro 10.000, C.P.6192, 13084-971 Campinas, São Paulo Brazil; Programa de Pós-graduação em Biologia Funcional e Molecular, Instituto de Biologia, Universidade Estadual de Campinas, Campinas, São Paulo Brazil; Programa de Pós-graduação em Genética e Biologia Molecular, Instituto de Biologia, Universidade Estadual de Campinas, Campinas, São Paulo Brazil; Instituto de Biologia, Departamento de Bioquímica e de Biologia Tecidual, Universidade Estadual de Campinas, Campinas, SP Brazil; Universidade Estadual de Campinas, Faculdade de Ciências Farmacêuticas, Campinas, São Paulo Brazil

**Keywords:** Neks, Interactomics, DNA damage response, Apoptosis, mRNA processing

## Abstract

**Background:**

Neks are serine-threonine kinases that are similar to NIMA, a protein found in *Aspergillus nidulans* which is essential for cell division. In humans there are eleven Neks which are involved in different biological functions besides the cell cycle control. Nek4 is one of the largest members of the Nek family and has been related to the primary cilia formation and in DNA damage response. However, its substrates and interaction partners are still unknown. In an attempt to better understand the role of Nek4, we performed an interactomics study to find new biological processes in which Nek4 is involved. We also described a novel Nek4 isoform which lacks a region of 46 amino acids derived from an insertion of an *Alu* sequence and showed the interactomics profile of these two Nek4 proteins.

**Results and discussion:**

Isoform 1 and isoform 2 of Nek4 were expressed in human cells and after an immunoprecipitation followed by mass spectrometry, 474 interacting proteins were identified for isoform 1 and 149 for isoform 2 of Nek4. About 68% of isoform 2 potential interactors (102 proteins) are common between the two Nek4 isoforms. Our results reinforce Nek4 involvement in the DNA damage response, cilia maintenance and microtubule stabilization, and raise the possibility of new functional contexts, including apoptosis signaling, stress response, translation, protein quality control and, most intriguingly, RNA splicing. We show for the first time an unexpected difference between both Nek4 isoforms in RNA splicing control. Among the interacting partners, we found important proteins such as ANT3, Whirlin, PCNA, 14-3-3ε, SRSF1, SRSF2, SRPK1 and hNRNPs proteins.

**Conclusions:**

This study provides new insights into Nek4 functions, identifying new interaction partners and further suggests an interesting difference between isoform 1 and isoform 2 of this kinase. Nek4 isoform 1 may have similar roles compared to other Neks and these roles are not all preserved in isoform 2. Besides, in some processes, both isoforms showed opposite effects, indicating a possible fine controlled regulation.

**Electronic supplementary material:**

The online version of this article (doi:10.1186/s12953-015-0065-6) contains supplementary material, which is available to authorized users.

## Background

Neks (NIMA-related kinases) are a group of serine-threonine kinases that are related to NIMA, their ortholog from *Aspergillus nidulans*, which is essential for cells to enter in mitosis. Human Neks show around 40% amino acid sequence similarity in their kinase domains with NIMA. While in *Aspergillus nidulans* there is only one NIMA, in humans there are eleven proteins that constitute the Nek family and that diverge in their N-terminal and specially C-terminal regulatory domains from NIMA [1,2]. For this reason, it has been speculated that human Neks show additional and diversified biological functions besides cell cycle control [3]. Until recently the only in depth studied Neks were Nek1, 2, 6, 7 and 9. All of these, except Nek1, are related to mitosis progression and the regulation of centrosome separation [4-6]. Nek1 has been described to be involved in the primary cilia formation [7,8], DNA damage response [8-11] and recently in apoptosis signaling [3,12,13].

Nek4, initially named as STK2 [14], is one of the largest human Nek proteins, constituted by an N-terminal kinase domain and a C-terminal regulatory domain. The human Nek4 gene is located on chromosome 3p21.1 and is transcribed into a ~4 kb mRNA, encoding an 841 residues protein [15].

The biological role of Nek4 is still not well understood. Some reports have already excluded the importance of Nek4 for cell cycle control [16,17] and others have demonstrated that Nek4 can display other functions, such as regulation of microtubule stability, primary cilium assembly, and association to replicative senescence and DNA damage response [16-18] shown also by other Neks, mainly, Nek1, Nek8 and Nek11 [9-11,19-22].

In an attempt to better characterize the Nek4 protein interactome and its possible functions, we obtained its cDNA from the human cell line HEK293T and performed a Nek4 immunoprecipitation followed by mass spectrometry (IP-MS) assay. We report here new insights into Nek4 functions, including its novel isoform, amplified by us. We also describe functional assays that reinforce Nek4 involvement in the DNA damage response and show for the first time an unexpected difference between both Nek4 isoforms in RNA splicing control.

## Results and discussion

### Identification of a novel Nek4 isoform

Nek4 was initially identified by Cance and co-workers [14] as STK2, from Serine/Threonine kinase 2, in a study using a kinase specific cDNA library from human breast cancer tumors or breast cancer cells. In that study they observed that STK2 showed homology to *Aspergillus nidulans* NIMA protein and its expression was observed at widely variable levels in human breast tumors. Later, Levedakou and co-workers [15] also isolated STK2 from a breast cancer cell line. Additionally, Levedakou and co-workers characterized the STK2 cell cycle expression profile as well as its tissue specificity, showing that this kinase is expressed in high levels, but not exclusively, in the heart and its mRNA levels are not cell cycle-dependent. After studies with murine STK2 [23,24], these proteins started to be renamed correctly as Nek4.

**Figure 1:**
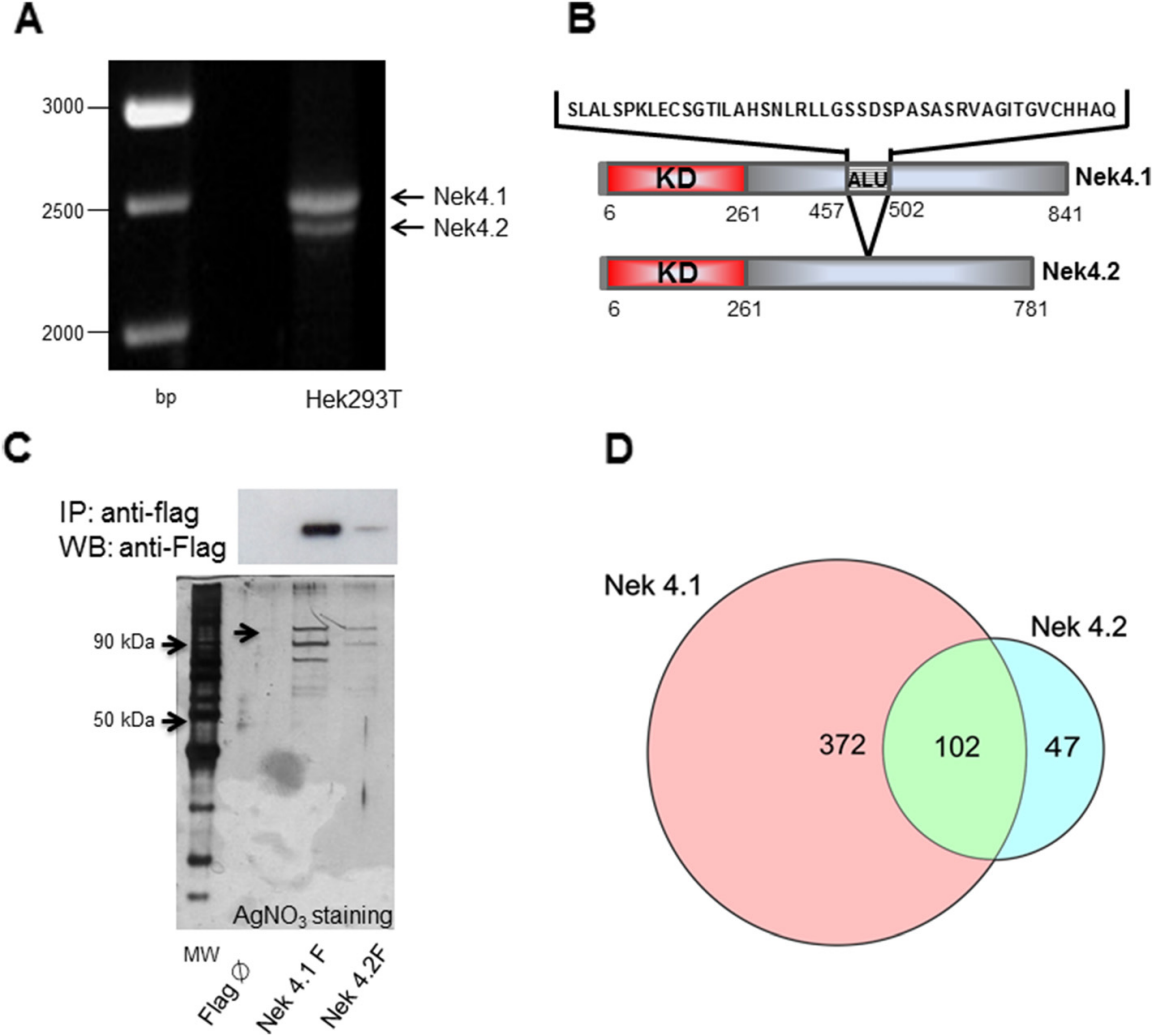
**Identification and characterization of two Nek4 isoforms. (A)** cDNAs to Nek4 isoform 1 (Nek4.1) and Nek4 isoform 2 (Nek4.2) were amplified from HEK293T cells extracts. **(B)** cDNA to Nek4.2 encodes a protein of 781 residues which is very similar to Nek4.1 except for the regulatory domain where Nek4.1 shows a 46 amino acid residues long insertion, which is encoded by an Alu DNA sequence. **(C)** HEK293 Flp-In T-REx stable cell lines expressing cDNA for both isoforms of Nek4 fused to a Flag tag were lysed and an immunoprecipitation for the Flag tag was performed. The eluate was used for mass spectrometry and western blotting analyses. Silver staining to Nek4 isoforms and immunoprecipitated proteins (below). Western blot to identify Nek4 isoforms (upper panel). **(D)** Venn diagram showing exclusive or common interactors of both Nek4 isoforms. Bp: DNA ladder. MW: Protein ladder. KD: kinase domain (red). ALU: translated region from retrotransposonAlu. Flag φ: empty vector – negative control.

In our study, we amplified the coding sequence (CDS) for a novel Nek4 isoform. The CDS for this isoform was amplified from cDNA libraries (data not shown) and also from HEK293T cells (Figure 1A). From the later, we also amplified the CDS for isoform 1 [GenBankRefseq: NM_003157.4], described by Levedakou and co-workers [15], which will be named as Nek4.1 in this report (Figure 1B, protein domains scheme). The novel amplified isoform [GenBank accession: KJ592714] is very similar to murine Nek4 long isoform [24] and we will denominate it here as Nek4.2. The difference between Nek4.1 and Nek4.2 is that the codifying cDNA for Nek4.1 contains a 138 bp insertion in the regulatory domain in comparison with Nek4.2 and also shows higher expression levels in HEK293 cells, suggesting that it is the predominant form (Figure 1A) [25]. This insertion corresponds to an *Alu* sequence, the most abundant DNA repetitive element in the primate genome. It was denominated *Alu* because it contains a sequence to *Alu*I restriction enzyme. Although the presence of this element in transcript regions is not common, growing evidences have suggested that transposable elements are found in coding sequences of up to 4% of human genes and that *Alu* elements correspond to 33% of these insertions [26,27]. The insertion of an *Alu* sequence could add a new splicing site in Nek4 mRNA, changing its expression profile. Besides, the translated protein from the mRNA containing the *Alu* sequence could have different functions and/or subcellular localization due to this potentially new interacting region [28,29]. In the case of Nek4, the *Alu* insertion does not promote a frame shift, encoding therefore a protein with additional 46 amino acid residues in the middle of its regulatory domain (Figure 1B).

As the information about Nek4 biological functions and protein interactome is scarce, we performed an IP-MS experiment with a stable cell line for Nek4 isoforms expression (Figure 1C) to obtain insights in the biological context in which this protein is involved. Interestingly, Nek4.2 showed about 68% of its potential interactors (102 proteins) in common with Nek4.1. Moreover, the number of partners identified for Nek4.1 (474) was three times greater than the obtained for Nek4.2 (150) (Figure 1D), pointing out that Nek4.1 may execute other functions in the cell, mediated by different interaction partners (Additional file 1).

On the other hand, the difference in the number of interaction partners found among both Nek4 isoforms may also be attributed to different levels of protein expression. Nek4.2 mRNA or protein could be less stable and, in this way, a smaller number of interactors were identified compared to Nek4.1. In Nek4, the *Alu* sequence was recently acquired, since both Nek4 isoforms are described for other genera from the *Hominidea* family, such as the chimpanzees (*Pan troglodytes* and *Pan paniscus*) and orangutans (*Pongo abelii*) but not for gorillas. For gorillas, such as for the more distant families like *Hylobatidae*, the only described coding sequence for Nek4 in the GeneBank is that corresponding to Nek4.2, indicating that this is most probably the ancestral isoform. Novel splice variants are initially free from selection pressure, as long as the original gene or mRNA remains functional and the variant is not disadvantageous. The novel splice variant can eventually become the constitutively spliced form [29]. For this reason, another possible interpretation for the higher number of interactions for Nek4.1, would be that isoform 1 could be the novel splice form, which has acquired new interactors and possibly new functions in comparison to Nek4.2.

### Interactomics suggests Nek4 involvement in different biological contexts

**Figure 2:**
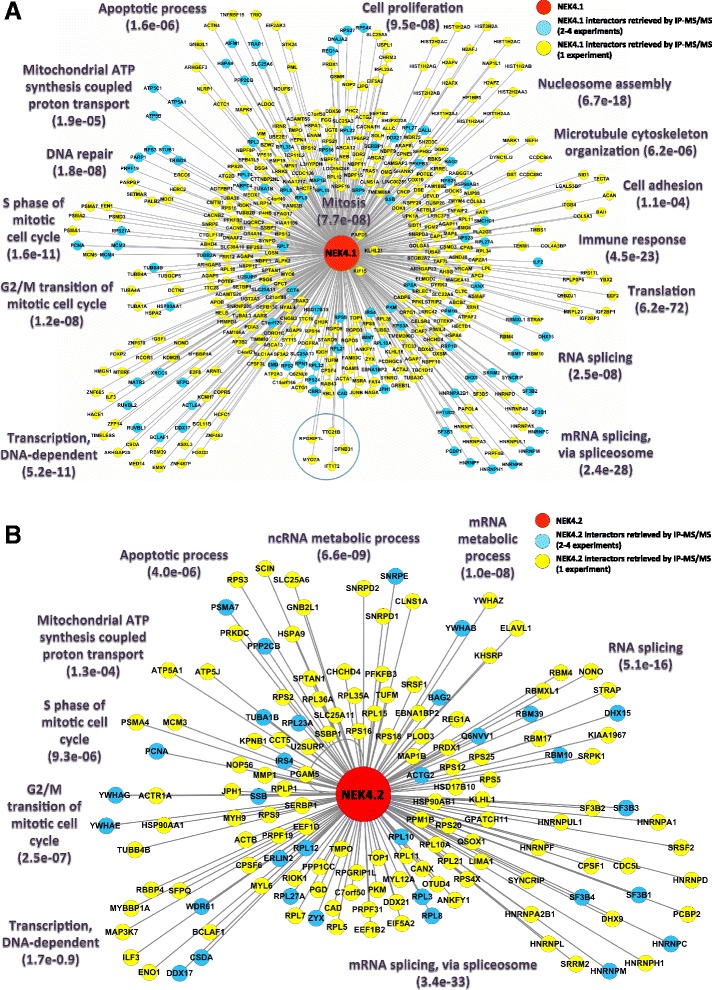
**Interaction networks of the retrieved Nek4.1 and Nek4.2 interactors from IP-MS.** The selected most relevant enriched GO biological processes are depicted in **(A)** Nek4.1 and **(B)** Nek4.2 networks by clustering the proteins involved in each of the biological processes with a circle layout. Clusters were assigned only to enriched biological processes containing at least two proteins (biological processes of specific cell types were not considered); proteins belonging to more than one biological process were assigned to clusters with the best enrichment p-values. More specific biological processes are shown only for proteins with more specific annotation in GO database. The red nodes correspond to both Nek4 isoforms, the cyan nodes correspond to proteins identified in more than 2 IP-MS biological replicates (from 2 to 4 experiments) and yellow nodes correspond to proteins identified in only one biological replicate. In (A) Nek4.1 network, TRAP1 was assigned to “Apoptotic process” according to Montesano and co-workers [30]; and 5 proteins involved in different cilium functions are depicted inside a blue circle: DFNB31, IFT172, MYO7A, RPGRIP1L and TTC21B [17,31-34]. The protein-protein interaction networks were built using the IIS platform and visualized using the Cytoscape software.

The Nek4 interactome shows proteins annotated in different cellular compartments that are also involved in biological processes already described for Nek4 or other Nek family members. On the other hand, it also shows Nek4 in completely new functions not described before (Figure 2).

The *in silico* PPI (protein-protein interactions) automated analysis and manual annotation (e.g. cilium maintenance/assembly) of Nek4.1 functions shows 14 enriched biological processes. Nek4.2 shows 9 enriched different biological processes and among these, 3 processes – mRNA splicing, apoptosis and cell cycle checkpoint – were also enriched in Nek4.1 analyses. Interestingly, mRNA splicing and RNA metabolism in general, are functional contexts common to both isoforms that were not discovered for either Nek4 or other Nek family members, until very recently, when this function was assigned for Nek2 [35]. Please see more details on this below.

Notably, some interesting Nek4 binding partners already described in the literature were also identified in our experiment. These include proteins such as: Fanton (RPGRIP1L) [17] and X-ray repair cross-complementing protein 6 (XRCC6) [18] or Ku70, thereby validating our results.

The identified Nek4 interacting proteins are localized in several cellular compartments, such as nucleus, mitochondria and endoplasmic reticulum. Nek4 shows a nuclear localization sequence (RRQKRREQTE) (NLS) in its regulatory domain with a high prediction score (NucPred predictor [36]), which has already been described previously [24]. The following cellular localization experiments, fractionation and functional assay were performed to analyze the interactions identified in the IP-MS experiment. In the next sections we will explore four different biological contexts that Nek4 could be involved in according to its protein interaction profile.

### Primary cilium functions

Human ciliopathies arise from defects in the primary cilium and can lead to obesity, retinal degeneration, polycystic kidney disease (PKD) and are also associated with a wide range of other morphological abnormalities [37]. Nek4 has been functionally implicated in the regulation of primary cilium stability, which was already described to other Nek family members, Nek1 and Nek8 [7,20]. Coene and co-workers showed that Nek4 interacts with RPGR-interacting protein 1 (RPGRIP1) and RPGRIP1-like protein (RPGRIP1L) [17], both associated with ciliopathies. After Nek4 knockdown, the number of ciliated cells decreases. However, this effect is not related to either RPGRIP1 or RPGRIP1L phosphorylation, suggesting Nek4 may act rather like a scaffold in the context of these cilia signaling proteins [17].

**Figure 3:**
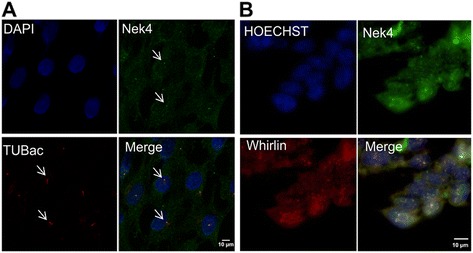
**Nek4 is localized at the base of the primary cilia. (A)** Nek4 colocalizes with Acetylated tubulin at the base of the cilium. RPE-hTERT cells were seeded on coverslips at high confluence and after 20 h of serum starvation were iced-cold methanol fixed. RPE-hTERT cells showed primary cilia induction which can be observed by mouse anti-Acetylated tubulin (a primary cilia marker) stain, and Nek4 colocalizes with acetylated tubulin at the base of the cilium (arrowhead). The nucleus was stained with DAPI. The images were acquired in HC PL APO CS2 63x/1.40 with OIL objective. **(B)** Nek4 shows a similar localization pattern as its putative interactor Whirlin. HEK293 cells were seeded on Cell Carrier 384 well plates coated with poly-L-lysine (Perkin Elmer), incubated for two days at 37°C with 5% CO2, then fixed with methanol and stained with proper antibodies. The nuclei were stained with Hoechst. Images were acquired with the Operetta automated microscope (Perkin Elmer) in non-confocal mode using the 60x high NA objective (NA = 0.9). The datasets were imported into Volocity for colocalization analysis and contrast correction. The images are representative of at least 10 replicate experiments.

The same group also showed the localization of Nek4 at the cilia root, suggesting that Nek4 could be important to cilia stability. The stability of cilia mediated by Nek4 could be related to microtubule stabilization, since Nek4 involvement in microtubule polymerization has already been shown by Doles and Hemann [16]. Indeed we found Nek4 to localize in a dotted fashion at the base of the primary cilium on RPE hTERT ciliated cells (Figure 3A) and we were able to find RPGRIP1L in our IP/MS assay validating our experiment. Besides RPGRIP1L, we have identified the proteins Whirlin (DFNB31) and Myosin-VIIa (MYO7a), in our IP/MS experiments among the Nek4.1 interactors. Both of these proteins are members of the Usher network, a complex of adapter and motor proteins, cellular adhesion factors and transmembrane receptors, which are important for cilia and stereocilia function and general cellular morphology [31,38-40].

Using immunofluorescence analysis we could also observe the same localization pattern for Nek4 and Whirlin (Figure 3B). Wright and co-workers showed that Whirlincolocalizes with RPGR^ORF15^, a splicing variant of retinitis pigmentosa GTPase regulator (RPGR), in photoreceptor connecting cilia [37]. In this context we have also identified two proteins involved in intraflagellar transport: Tetratricopeptide repeat protein 21 B (TTC21B) and Intraflagellar transport protein 172 homolog (IFT172) [32,33]. As we identified here different proteins important for cilia function, we believe that Nek4 could interact with a complex of proteins and, among them, some could also be Nek4 substrates. Clearly, more studies are necessary to better understand Nek4 role in this context and to identify its possible substrates.

### Mitochondria-related functions

In our IP-MS experiment we have identified several proteins related to the apoptotic process (Figure 2), such as Heat shock protein 75 kDa (TRAP1), Apoptosis-inducing factor 1 (AIFM1) and ANT3 (SLC25AC). ANT3 is an ADP/ATP translocator which could also be part of the mitochondria permeability transition pore (PTP) complex and may therefore participate in apoptosis [41,42]. Previously, another Nek family member, Nek1, was related to an anti-apoptotic mitochondrial-driven process [12]. In that study, the authors found that Nek1 phosphorylates the membrane protein VDAC, another component of the PTP, in the cytoplasmic domain, thereby preventing the channel opening and release of Cytochrome c, required to initiate apoptosis.

**Figure 4:**
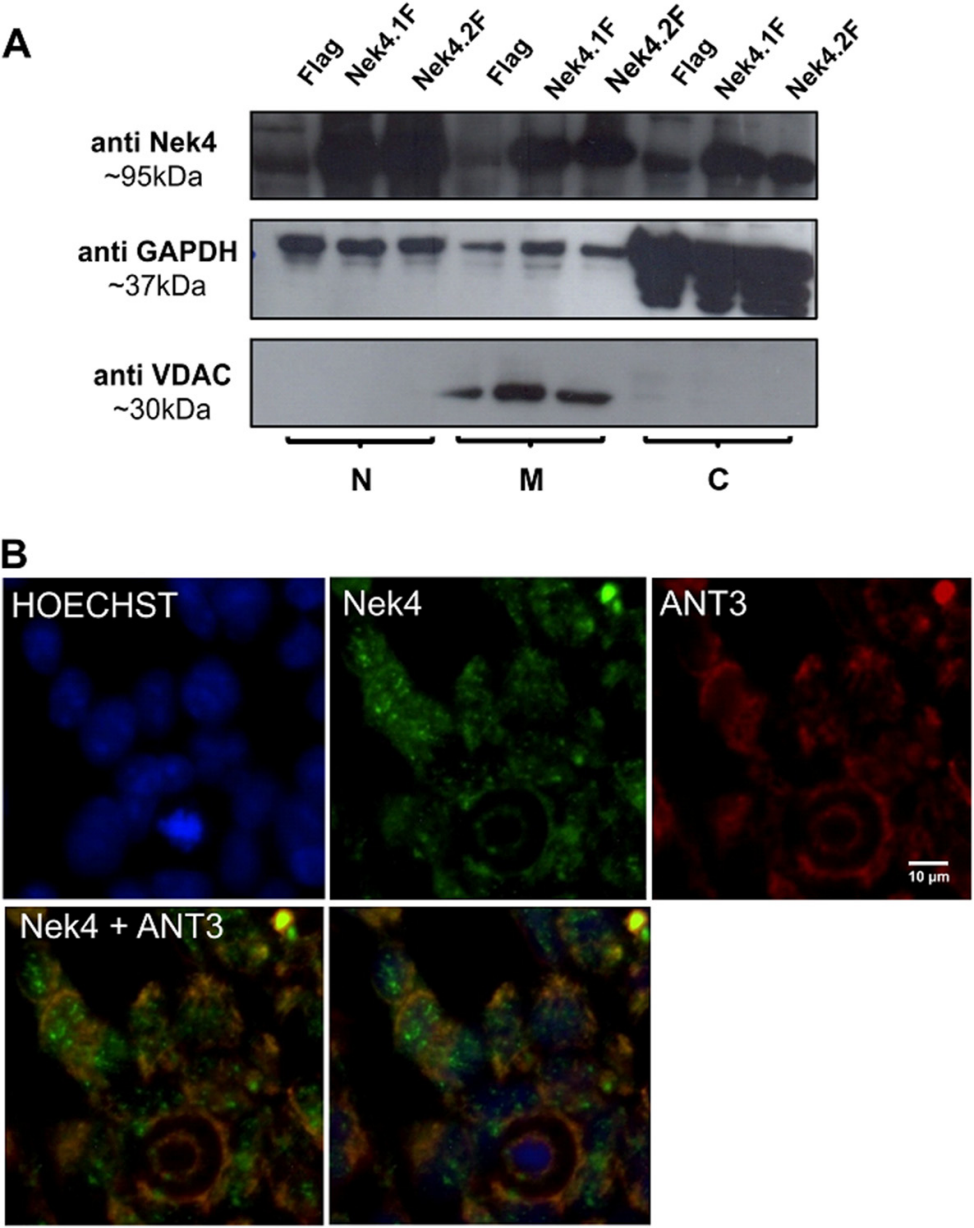
**Nek4 copurifies with the mitochondrial fraction. (A)** Nek4 is present in mitochondrial fractions and shows a similar perinuclear pattern of localization **(B)** such as its putative interactor ANT3, a mitochondrial protein. HEK293 cells were seeded on Cell Carrier 384 well plates coated with polylysine (Perkin Elmer), incubated for two days at 37°C with 5% CO2, then fixed with methanol and stained with proper antibodies. The nuclei were stained with Hoechst. Images were acquired with the Operetta automated microscope (Perkin Elmer) in non-confocal mode using the 60x high NA objective (NA = 0.9). The datasets were imported into Volocity for colocalization analysis and contrast correction. The images are representative of at least 10 replicate experiments. The mitochondrial localization of Nek4 was verified by cell fractioning of HEK293 Flp-In (stably expressing Nek4 isoforms or empty vector - Flag). Using Qproteome Mitochondria Isolation Kit (QIAGEN). N: nuclear, M: mitochondrial and C: cytosolic fractions.

Most of these identified proteins are localized to mitochondria suggesting that Nek4 may be present in this cellular compartment or regulate some of its protein components. To verify this possibility we performed a cellular fractionation experiment and indeed, we could observe Nek4 presence in mitochondrial fractions (Figure 4A). To further analyze the presence of Nek4 in mitochondria, we performed a subcellular localization assays with the mitochondrial protein and Nek4 interaction partner ANT3 (Figure 4B). Nek4 was predominantly found in the nucleus while ANT3 showed a perinuclear staining consistent with its posssible mitochondrial localization. While these two proteins did not colocalize significantly, the subcellular fractionation strongly suggests that at least part of Nek4 is associated with mitochondria.

### DNA damage response

Recently, Nguyen and co-workers suggested a role for Nek4 in the DNA damage response (DDR) [18]. In that study, Nek4-depleted cells were found to be resistant to enter in replicative senescence and also to agents that induce DNA double-breaks (etoposide, bleomycin and γ-irradiation). This is probably due to a decrease of γH2AX activation, which may result from an impaired recruitment of DNA-PKc, Ku70 or Ku80, all essential to the Non-Homologous End Joining response (NHEJ). The latter three proteins were identified in that study as interactors of Nek4 in IP-MS experiments.

**Figure 5:**
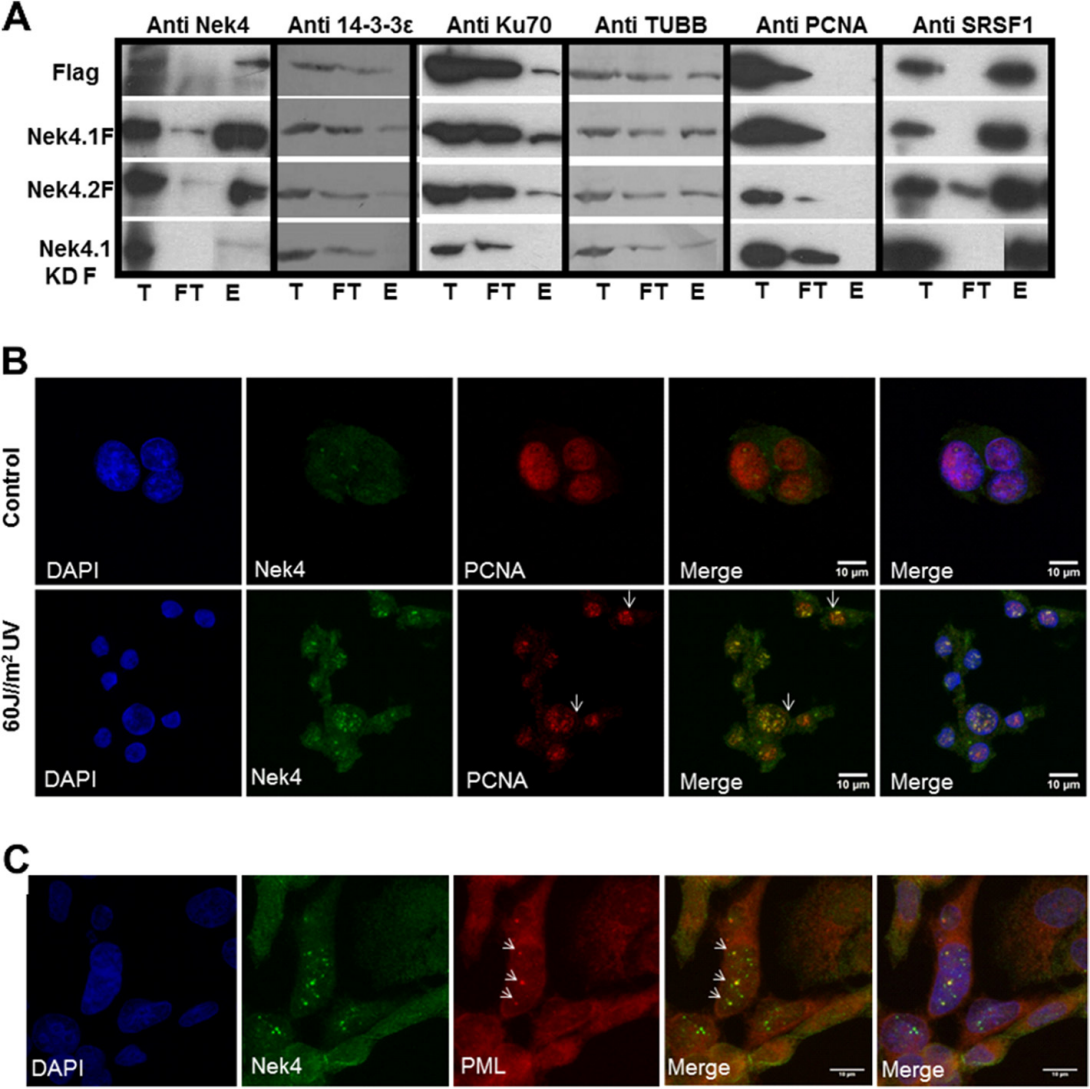
**Nek4 interacts and colocalizes with DNA damage response proteins. (A)** Cells expressing empty vector (Flag), isoform 1 (Nek4.1 F), isoform 2 (Nek4.2 F) or kinase dead mutant of Nek4.1 were lysed and phosphoproteins present in these cells were purified. **(B)** Nek4 and PCNA co-localization is UV exposition-dependent. HEK293T cells were or not (control) UV irradiated (60 J/m^2^). One hour after irradiation, the cells were methanol fixed and indirect immunofluorescence against Nek4 and PCNA was performed. DNA was stained with DAPI. Only irradiated cells showed a strong Nek4 and PCNA colocalization. **(C)** Dotted Nek4 and PML localization show partially overlapping patterns. The image presented here is a section from the original image to emphasize the PML bodies. Two fields were analyzed and from these 14 cells with positive staining for PML bodies were observed. Arrows show that some dotted staining of Nek4 corresponds to PML bodies. Seven cells showed nuclear dots to Nek4 and PML. In these nuclei 62% of PML bodies showed positive colocalization with Nek4, and, 27% of Nek4 dot like staining showed positive colocalization with PML bodies. Thus, the partial colocalization of Nek4 with PML indicates that most of PML bodies contain Nek4, but, Nek4 is present also in other nuclear structures in addition to PML bodies. Nek4 and PML stained dots were manually counted using FIJI software. The images were acquired in HC PL APO CS2 63x/1.40 with an OIL objective. T: total, FT: flow through, E: eluate.

We have also found Ku70 (XRCC6) among Nek4.1 interacting partners (Figure 2). Although we could not confirm the interaction through pull-down and Western Blot (Additional file 2), when we purified phosphorylated proteins from cells expressing both Nek4 isoforms or a kinase dead mutant we observed that the amount of phosphorylated Ku70 in Nek4.1 kinase dead expressing cells is less than in the wild type Nek4 protein expressing cells (Figure 5A, Anti Ku70). This result suggests that Ku70 and Nek4 interaction may be transient and possibly dependent on the Nek4 auto-phosphorylation status or on the Nek4 dependent phosphorylation status of Ku70.

Another interactor in the repair context is PCNA, a component of the Nucleotide Excision Repair (NER) pathway [43]. NER is activated in response to pyrimidine dimer formation, usually caused by UV radiation or cisplatin adducts, among other stimuli [44]. Although, we could not confirm neither the presence of PCNA in immunoprecipitates from Nek4 expressing cells (Additional file 2) nor in pre-purified phosphorylated proteins (Figure 5A, Anti-PCNA), the interaction may occur in a specific cellular situation and compartment. This hypothesis was confirmed after we exposed cells to UV irradiation. In non-irradiated cells PCNA showed a diffuse nuclear localization, but, after UV irradiation, PCNA was concentrated at damage foci and clearly colocalized with Nek4 (Figure 5B). This result suggests Nek4 involvement in DDR and in NER, besides NHEJ pathway.

Furthermore, we found a partial co-localization (30%) of Nek4 with PML bodies (Figure 5C), structures involved in the storage of DNA repair proteins. The number of the PML bodies increases after IR-induced DNA damage [45]. The partial Nek4 localization in these structures suggests that Nek4 interacts with DNA repair proteins under specific conditions.

### mRNA processing

**Figure 6:**
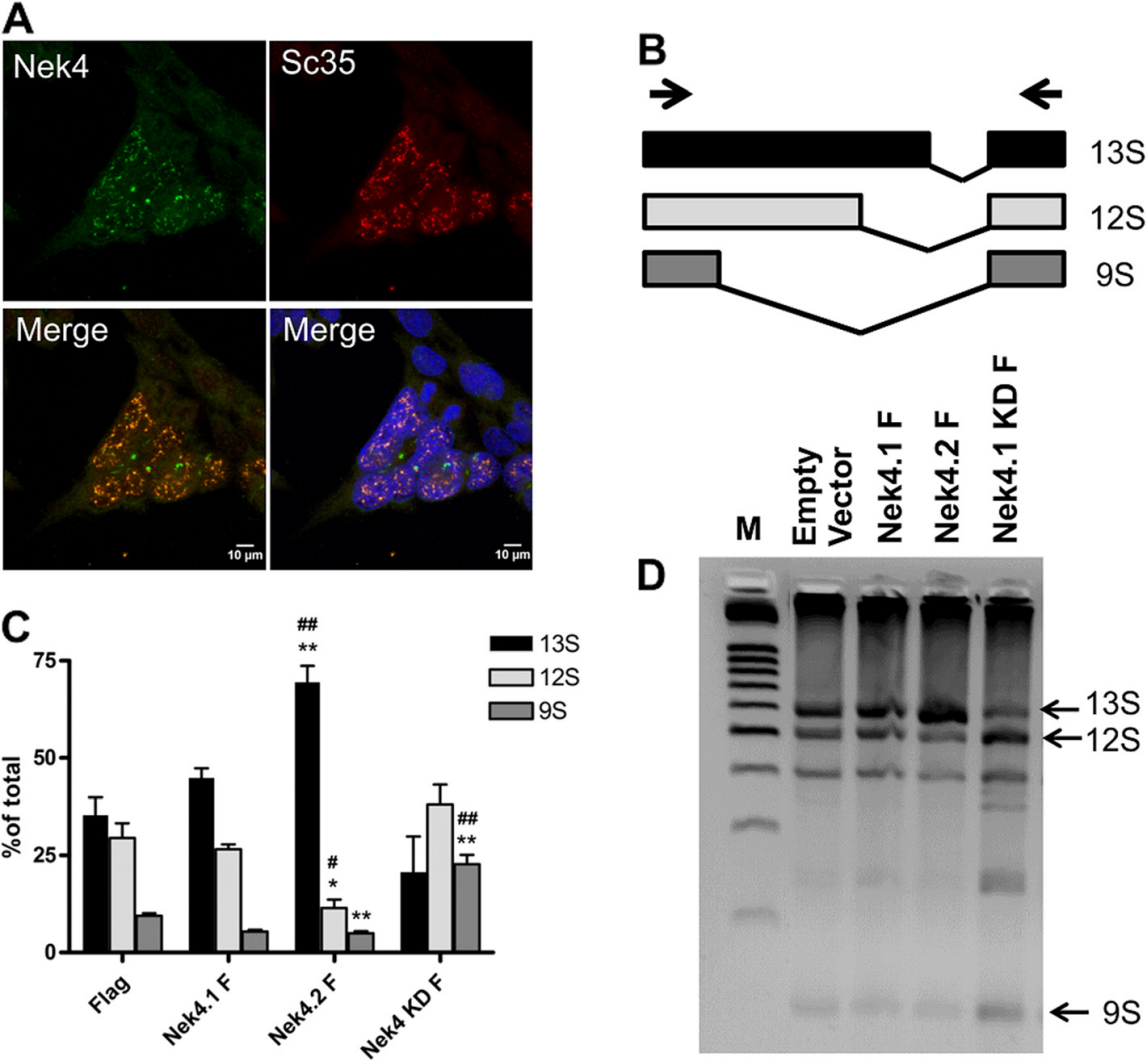
**Nek4 participates in mRNA splicing regulation.** HEK293T cells were methanol fixed and immunofluorescence against endogenous Nek4 and SC35 (a nuclear speckle marker) was performed. **(A)** Confocal microscopy shows the dotted staining of Nek4 in the nucleus, related to nuclear speckles (colocalization with SC-35). **(B-D)** To verify the involvement of Nek4 in RNA splicing, HEK293 Flp-In T-REx cells stably expressing empty vector (Flag), Nek4.1 F, Nek4.2 F or Nek4.1 KDF were transfected together with the E1A minigene encoding the pMTE1A plasmid. After 48 h, total RNA was extracted and the cDNA synthesized. Minigene splicing generates 3 main variants (represented in B), depending on the splicing site selection. The E1A isoforms generated in Nek4.1, Nek4.2 and Nek4.1 KD expression can be visualized in the agarose gel, depicted in **(D)**. The band intensities for each isoform were measured and the percentage of 9S, 12S and 13S isoform in relation to the total isoforms generated is depicted in **(C)**. Vertical bars in the graphs indicate ± standard deviation. Wherever it exists, the significance of the difference relative to the control (empty vector - Flag) is indicated by *p < 0.05.**p < 0.01. # represents the significant difference relative to Nek4.1. # p< 0.05 and ## p < 0.01; n = 3 (t – Test).

Our protein-protein interaction network indicated a possible involvement of Nek4 in mRNA processing, especially spliceosome mediated processing (Figure 2). Interestingly, our immunofluorescence data further show endogenous Nek4 localization to nuclear speckles (Figure 6A), which are nuclear substructures known to be enriched in small nuclear ribonucleoprotein (snRNP) and many other transcription and pre-mRNA splicing related proteins (reviewed in [46]).

Therefore, we investigated whether Nek4 could modulate the splicing site selection of the adenoviral E1A minigene, a common assay to verify *in vivo* splicing regulation. Depending on the 5’-splice site selection, the E1A pre-mRNA may generate five isoforms: 13S, 12S, 11S, 10S, and 9S (Figure 6B) [47]. The 9S, 12S and 13S isoforms can be easily monitored by RT-PCR followed by agarose gel analysis, where the intensity of each band in the gel directly correlates with the splicing site selection, which, in turn, reflects the positive or negative influence of regulatory proteins [47]. Cells stably expressing Nek4.1 do not show shift of splicing site selection related to the negative control, but, remarkably, cells expressing Nek4.2 show a preference to more distal splicing sites, leading to the formation of the 13S isoform, with a concomitant reduction in 9S and 12S isoforms formation (Figure 6C and D). On the other hand, cells expressing the kinase dead mutant of Nek4.1 show an opposite effect, with an increase in 9S formation (Figure 6C and D). This difference observed between both isoforms may be attributed to differences among their interaction partners. While both Nek4 isoforms interacted with hNRNPA1, just isoform 2 interacted with SRSF1, SRSF2 and SRPK1 (Additional file 1), all important regulators of RNA splicing and frequently associated to the switch from the 13S to the 9S 5’ splice preference [48]. Moreover, this result could indicate a cross-regulation between Nek4 isoforms, with a dominant negative interference effect mediated by the Nek4 kinase dead mutant in this regulation.

During this manuscript preparation, Naro and co-workers described the involvement of Nek2 on RNA splicing and in their study they found that Nek2 phosphorylates SRSF1 and modulates RNA splicing of apoptotic proteins [35]. These results support our findings which included a novel function to Neks family.

## Conclusions

Here we have characterized the interaction profile of Nek4 and its novel isoform. These results allow the confirmation and extension of the current knowledge on the biological processes in which Nek4 participates. This study shows that the novel isoform, Nek4.2, is involved in mRNA processing, apoptosis and transcription, all processes not yet explored for other Nek family members. Notably, Nek4.1, which contains an insertion in its regulatory domain, shares interactions and biological processes with isoform 2 but also holds functions already described for other Neks, such as DNA repair and cilia processes, and even other new functions, such as regulation of translation and cell adhesion, previously not described for Nek family members.

Our functional results reinforce Nek4 involvement in the DNA damage response, suggesting roles in different types of repair pathways, and, moreover, show an unexpected difference between both isoforms in RNA splicing control.

A better understanding of the Nek4 interactions shown here in its biological context requires an in-depth and detailed investigation. However, our findings are the first steps towards the elucidation of novel Nek4 roles and consist in the pieces of a big puzzle that need to be integrated. Further studies are necessary to dissect the real role of Nek4 in these novel functions described here, such as mRNA processing, apoptosis and also the connection between these processes.

## Methods

### Molecular cloning

The full length Nek4 [GenBankRefSeq: NM_003157.4] and its novel isoform sequence [GeneBank accession: KJ592714], named here as Nek4.1 and Nek4.2, respectively, were amplified by PCR from a human fetal brain cDNA library (Clontech) and cloned into the pcDNA5-FRT-TO vector between *BamH*I and *Not*I restriction sites. The occurrence of these isoforms was confirmed by RT-PCR amplification from HEK293 cells.

### Cell culture and establishment of a stable cell line

HEK293T, HEK293 Flp-In T-REx® and HeLa cell lines were maintained in Dulbecco’s modified Eagle’s medium (DMEM, GIBCO) plus 10% FCS, supplemented with 2 mM L-Glutamine and 100 U/ml penicillin-streptomycin. Stable cell lines to Nek4 expression were generated from HEK293 Flp-In T-REx cells (Invitrogen), containing recombination sites. These cells were transfected with pcDNA5–FRT-TO vector containing codifying sequences to both Nek4 isoforms expression or empty vector fused to a Flag tag and a recombinase expression plasmid (pOG44) using Lipofectamine (Invitrogen). After selection with hygromycin (100 μg/mL), the clones were tested for Nek4 expression, induced by addition of tetracycline (500 ng/ml) in culture medium, using Western blot analysis.

RPE-hTERT cells were kindly provided by Dr. Joan Roig and were cultivated in DMEM/ F12 medium supplemented with 10% of FCS. Primary cilium induction was performed by serum starvation (20 h) in high confluence cultures.

### Cell lyses and immunoprecipitation

In order to identify Nek4 binding partners, HEK293 Flp-In FRT cells were grown at 70% confluence in 175 cm^2^ flasks (5 flasks per condition) and then 500 ng/mL of tetracycline was added in culture medium. Cells were harvested 48 h after the induction with tetracycline. Pellets were washed with PBS and then centrifuged for 5 min at 450 g and resuspended in lysis buffer (50 mm Tris–HCl, pH 7.4, containing 150 mM NaCl, 1 mM EDTA, 1% Triton X-100) supplemented with protease inhibitor cocktail (Roche) and phosphatase inhibitors (2 mM Sodium Orthovanadate, 10 mM β-glycerophosphate, 1 mM Sodium Fluoride (Sigma)). After 30 min of incubation on ice, the cell lysates were centrifuged for 15 min at 12,000 g at 4°C to clear cell debris. Total protein concentration was determined by Bradford assay according to the manufacturer’s instruction (Sigma). Equal amount of cell lysates to each sample were incubated at 4°C overnight under gentle agitation with 150 μL of anti-FLAG M2 affinity gel (Sigma). The beads were washed three times with TBS buffer (150 mM Tris–HCl, pH 7.4 and 150 mM NaCl) and the complexes were eluted with 3x FLAG peptide (Sigma) at a final concentration of 150 ng/μL for 30 min at 4°C, under agitation. The eluate was analyzed by Western blot and mass spectrometry.

### Western Blot

Eluted proteins were separated by SDS-PAGE in sample buffer (250 mM Tris-HCl pH 6,8; 0,8% SDS; 0,2% bromo-phenol blue; 45% glycerol; 20% 2-β-mercaptoethanol) and then transferred onto a 0.45 μm PVDF membrane (Millipore). After transfer, the membrane was blocked with TBS-T (TBS containing 0.1% of Tween 20) containing 5% non-fat milk. The primary antibody used were mouse anti-Flag M2 (Sigma 1:5000), mouse anti-PCNA (Cell Signaling 1:2000), goat anti-Nek4 (Santa Cruz Biotechnology 1:500), rabbit anti-beta-tubulin (Abcam 1:1000), mouse anti-Ku70 (Thermo Scientific 1:500), rabbit anti-GAPDH (Santa Cruz Biotechnology 1:500), rabbit anti-14-3-3ε (Thermo Scientific 1:5000), mouse anti-VDAC (Abcam 1:1000), mouse anti-SF2 (SRSF1) (Invitrogen 1:500) and the membrane was probed overnight at 4°C under gentle agitation. After the washing step with TBS-T, the membrane was incubated for 1 hour with species-specific horseradish peroxidase-conjugated secondary antibody: anti-mouse (Calbiochem, 1:5000 dilution), anti-goat or anti-rabbit (Sigma, 1:5000 dilution) antibodies. Some membranes were probed with phosphatase alkaline-conjugated secondary antibody (anti-rabbit from Sigma at 1:3000 dilution). The immunoreactive protein signals were developed using Luminol (Santa Cruz Biotechnology) and the membranes were exposed to photographic film (GE Healthcare) or to alkaline phosphatase substrate (BCIP/.NBT from Sigma-Aldrich). In addition, the protein bands were visualized in SDS-PAGE using silver staining.

### LC-MS/MS

The immune complexes (80-200 μg of protein) were reduced (500 μM dithiothreitol for 30 min at 56°C), alkylated (4 mM iodoacetamide for 30 min at room temperature in the dark), and digested with trypsin (Promega). The samples were dried in a vacuum concentrator and reconstituted in 20 μL of 0.1% formic acid. 4.5 μL of the resulting peptide mixture was analyzed on an ETD enabled LTQ Velos Orbitrap mass spectrometer (Thermo Fisher Scientific) coupled with LC-MS/MS by an EASY-nLC system (ProxeonBiosystem) through a Proxeon nano electrospray ion source or an RP-nanoUPLC (nano Acquity, Waters) coupled with a Q-Tof Ultima mass spectrometer (Waters).

All of the instrument methods for the LTQ Velos Orbitrap were set up in the data-dependent acquisition mode. Peak lists (msf) were generated from the raw data files using Proteome Discoverer version 1.3 (Thermo Fisher Scientific) with Sequest search engine against Swiss-Prot human database (released March 25^Th^2013), with carbamidomethylation (+57.021 Da) as the fixed modification and methionine oxidation (+15.995 Da) as variable modification, allowing one trypsin missed cleavage site and a tolerance of 10 ppm for precursor and 1 Da for fragment ions. The msf files generated by Proteome Discoverer software were analyzed in ScaffoldQ + v.3.3.2 (Proteome Software), with scoring parameters adjusted to obtain a false discovery rate less than 1%.

In the search performed using Mascot engine, only peptides with a minimum of five amino acid residues which showed significant threshold (p < 0.05) in Mascot-based score were considered in the results. The MS/MS assays were performed by the Mass Spectrometry Facility at Brazilian Biosciences National Laboratory (LNBio), CNPEM, Campinas, Brazil.

### Mass spectrometry data analysis and *In silico* PPI analysis

To classify more reliable interactions, only exclusive proteins (no peptides in replicated Flag control samples) were considered. Some proteins already described in literature as Nek4 interaction partners were identified in only one replicate and, for this reason, proteins that were identified in only one replicate were also maintained on the subsequent analysis and were discriminated on Additional file 1 and Figure 2.

The retrieved Nek4.1 and Nek4.2 interacting partners from IP-MS were integrated in interaction networks using the Integrated Interactome System (IIS) platform, developed at National Laboratory of Biosciences, Brazil [49]. The enriched biological processes from the Gene Ontology (GO, http://www.geneontology.org/) database were calculated in each network using the hypergeometric distribution [49]. The interaction networks were visualized using Cytoscape 2.8.3 software [50].

### Indirect Immunofluorescence

In order to perform the image experiments, HeLa or HEK293T cells were grown in coverslips or on Cell Carrier 384 well plates coated with polylysine (Perkin Elmer) and fixed and permeabilized with ice cold methanol for 10 minutes at −20°C. Fixed cells were blocked in PBS-T (1X PBS, 0.1% Triton X-100, and 3% BSA) for 20 minutes and then incubated in the same buffer with primary antibodies: goat anti-Nek4 (SC5517), mouse anti-Nek4 (SC81332), mouse anit-whirlin (SC271939) or goat anti-PML (SC9863), from Santa Cruz Biotechnology, rabbit anti-14-3-3e (PA5-24165) or mouse anti-SLC25A6 (PA5-29199), from Thermo Scientific, and mouse anti-SC-35 (ab11826). Primary antibodies were detected in the same buffer for 20 minutes with secondary antibodies: donkey anti-goat Alexa Fluor 488, chicken anti-goat Alexa Fluor 488, chicken anti-mouse Alexa Fluor 546 or donkey anti-rabbit Alexa Fluor 546 from Invitrogen. DNA was stained with 4,6-diamidino-2-phenylindole (DAPI, 0.01 mg/ml) or Hoechst (0.06 mg/ml). Cells were visualized in non-confocal Operetta automated microscope (Perkin Elmer) or confocal Leica SP8, LSM780 NLO (Zeiss) using the 60x NA objective. The confocal acquisitions were performed at National Institute of Science and Technology on Photonics Applied to Cell Biology (INFABIC) or at LNBio. The images treatments were performed on Fiji software [51] or, when using Operetta microscope, the datasets were imported into Volocity for colocalization analysis and contrast correction.

### Mutagenesis and Nek4 kinase dead generation

Kinase dead form of Nek4.1 (Nek4KD) was generated with QuickChange™ Site-Directed Mutagenesis Kit (Agilent Technologies) according to the manufacturer's recommendations. Codifying DNA to Nek4 isoform 1 was used as template to generate kinase dead mutant (K35/36 M). The primers used were 5’ GCGGGACGGCAGCAGTATGTCATCATGATGCTGAACCTCCGAAATGCC 3’ and 5’ GGCATTTCGGAGGTTCAGCATCATGATGACATACTGCTTGCCGTCCCGC 3’ (reverse) and the mutation confirmed by DNA sequencing. Then, as described before, stable cells were generated to express Nek4 KD Flag construct.

### Phosphoprotein Purification

Phosphoproteins were purified from cells stably expressing the empty vector (Flag), Nek4.1 isoform, Nek4.2 isoform or Nek4KD mutant according to the manufacturer's instructions (Qiagen). Briefly, cells were lysed in the Lysis Buffer, and the cell lysate was centrifuged at 10,000 × g at 4°C for 30 min to remove insoluble material. After centrifugation, the protein concentration of the cell lysate was quantitated by Bradford assay, and an equal protein amount was applied to a Lysis Buffer-equilibrated phosphoprotein purification column at room temperature. The flow through representing non-phosphorylated proteins was collected and after washing the column with Lysis Buffer, the phosphoproteins were eluted with Phosphoprotein Elution Buffer. Equivalent amount of proteins were run in SDS-PAGE and a western blotting assay using specific antibodies was performed.

### Minigene E1A RNA splicing assay

For the *in vivo* splicing analysis, HEK293 Flp-In cells stably expressing an empty vector (Flag), Nek4 isoforms (Nek4.1 F or Nek4.2 F) or a Nek4 kinase dead (Nek4KD) were transiently transfected with the minigene E1A encoding plasmid pMTE1A [52]. After six hours of transfection the medium was changed and tetracycline was added to induce protein expression. The total RNA was extracted 48 h after the transfection with 1 mL of TRizol reagent (Life Technologies Corporation) according to the manufacturer’s protocol. cDNA synthesis was performed using oligo(dT) primer (Invitrogen) and the Moloney murine leukemia virus reverse transcriptase (Invitrogen) from 1 μg of total RNA. The PCRs were performed with the 5’-ATTATCTGCCACGGAGGTGT-3’(forward) and 5’-GGATAGCAGGCGCCATTTTA-3’ (reverse), as previously described [53]. After separation of the amplification products containing GelRed (Biotium) on 3% agarose gels the band intensities were calculated using the software imageJ [54]. The intensities of all isoforms were summed, set as 100%, and used to normalize the intensity of each band.

## Additional files

Additional file 1:
**Table of exclusive and common interactors of Nek4.1 and Nek4.2 isoforms.** The proteins identified by the IP-MS assays of Nek4.1 and Nek4.2 and not identified in the negative control (empty vector) are presented. Some proteins were identified for only one isoform and others were identified in immunoprecipitades of both isoforms. The number of experiments in which the proteins were found is shown. Additional file 2:
**Western blot analysis of proteins identified by the IP-MS experiments.** Cells expressing Nek4.1 F, Nek4.2 F, or empty vector (Flag) were lysed and proteins from the extracts were immunoprecipitated using anti-Flag antibody. Western blotting using specific antibodies against some of the proteins identified in IP-MS experiments was performed. T: total sample. IP: immunoprecipitated sample. As Nek4 is a kinase, it is expected that its interactions would be transitory and probably occur in a specific cellular condition. For this reason, the amount of its interaction partners in the immunoprecipitate does probably not allow its identification in a Western blot assay, less sensitive than mass spectrometry analysis.
