# Supplementary figures and images for: New interaction partners for Nek4.1 and Nek4.2 isoforms: from the DNA damage response to RNA splicing
Source: Proteome Sci. 2015 Feb 26;13:11. doi: 10.1186/s12953-015-0065-6 (PMC4367857; doi:10.1186/s12953-015-0065-6)

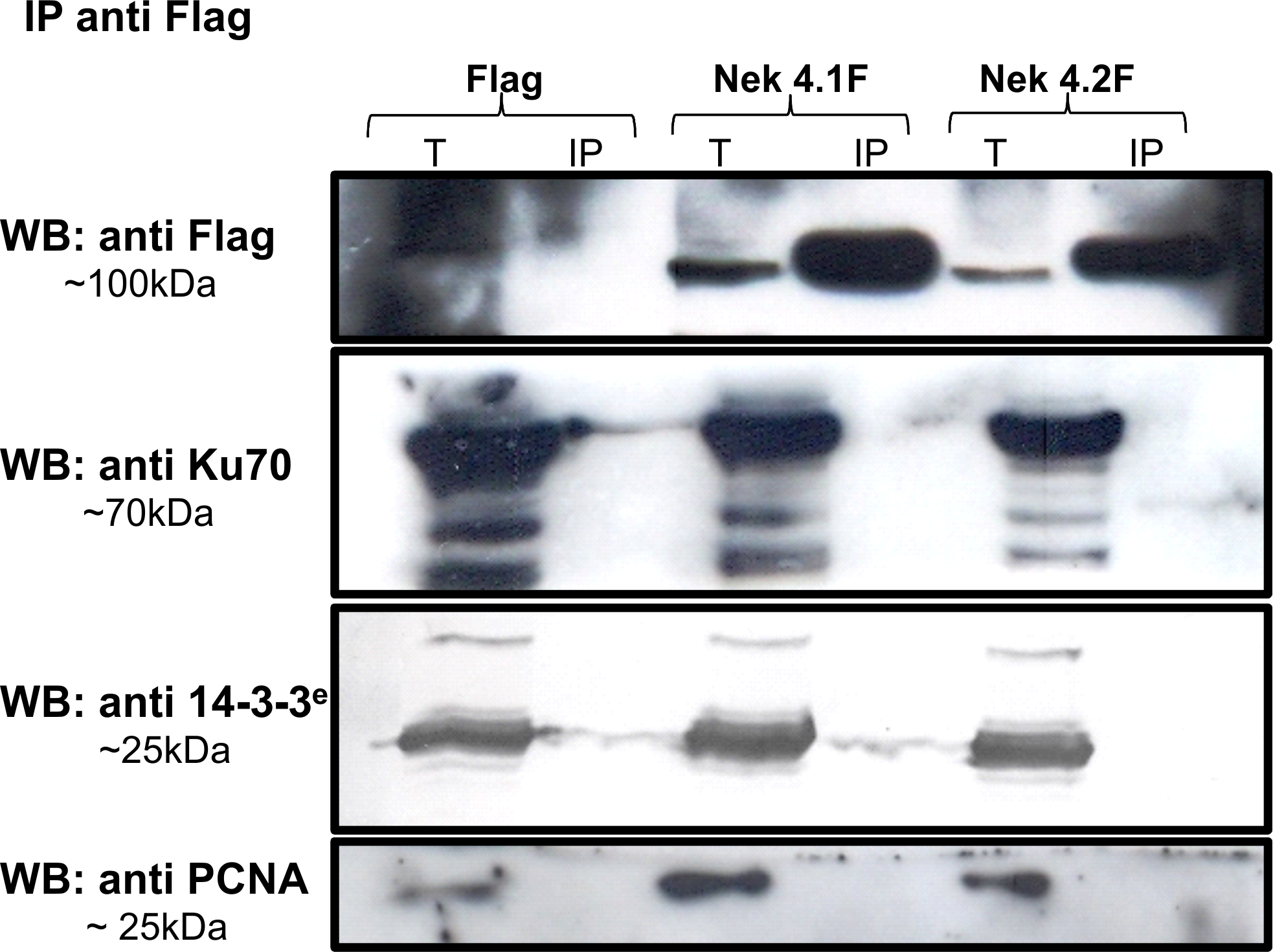

Supplement: Additional file 2: — Western blot analysis of proteins identified by the IP-MS experiments. Cells expressing Nek4.1 F, Nek4.2 F, or empty vector (Flag) were lysed and proteins from the extracts were immunoprecipitated using anti-Flag antibody. Western blotting using specific antibodies against some of the proteins identified in IP-MS experiments was performed. T: total sample. IP: immunoprecipitated sample. As Nek4 is a kinase, it is expected that its interactions would be transitory and probably occur in a specific cellular condition. For this reason, the amount of its interaction partners in the immunoprecipitate does probably not allow its identification in a Western blot assay, less sensitive than mass spectrometry analysis. [file 12953_2015_65_MOESM2_ESM.tiff]
